# Preliminary study on the potential impact of probiotic combination therapy on *Helicobacter pylori* infection in children using 16S gene sequencing and untargeted metabolomics approach

**DOI:** 10.3389/fmicb.2024.1487978

**Published:** 2024-10-31

**Authors:** Ya Yan, Lingjun Dong, Juan Xu, Zhijiao Zhang, Pengyan Jia, Jingmin Zhang, Weihong Chen, Weiqi Gao

**Affiliations:** ^1^Third Hospital of Shanxi Medical University, Shanxi Bethune Hospital, Shanxi Academy of Medical Sciences, Tongji Shanxi Hospital, Taiyuan, China; ^2^School of Pharmacy, Shanxi Medical University, Taiyuan, China; ^3^Shanxi Academy of Advanced Research and Innovation (SAARI), Taiyuan, China

**Keywords:** *Helicobacter pylori*, probiotic therapy, mechanism, 16S rRNA gene sequencing, untargeted metabolomics

## Abstract

**Objective:**

The purpose of this study was to explore the potential mechanism of *Helicobacter pylori* (Hp) eradication by probiotic therapy through 16S rRNA gene sequencing technology and untargeted metabolomics.

**Methods:**

Twenty four Hp-infected children were recruited from the Shanxi Bethune Hospital, and 24 healthy children were recruited as a blank control group. Group A: fecal samples from 24 healthy children. Group B: fecal samples of 24 children with Hp infection. Group B1 (*n* = 15): fecal samples of group B treated with probiotic therapy for 2 weeks. Group B2 (*n* = 19): fecal samples of group B treated with probiotic therapy for 4 weeks. The above fecal samples were analyzed by 16S rRNA gene sequencing technology and untargeted metabolomics.

**Results:**

There was no significant difference in alpha diversity and beta diversity among the four groups, but many bacteria with statistical difference were found in each group at the bacterial genus level and phylum level. LEfSe results showed that in group B, *Porphyromonadaceae*, *Shigella* and other microorganisms related to intestinal microecological dysbiosis were enriched. And in group B2, abundant characteristic microorganisms were found, namely *Bacillales* and *Prevotella*. KEGG metabolic pathway enrichment analysis showed that groups B1 and B2 were involved in 10 metabolic pathways potentially related to probiotic treatment: purine metabolism, nitrogen metabolism, arginine biosynthesis, alanine, aspartic acid and glutamate metabolism, glyoxylic acid and dicarboxylic acid metabolism, unsaturated fatty acid biosynthesis, fatty acid extension, fatty acid degradation, pyrimidine metabolism, fatty acid biosynthesis.

**Conclusion:**

Probiotic therapy can inhibit Hp to some extent and can relieve gastrointestinal symptoms, making it a preferred therapy for children with Hp infection and functional abdominal pain. Hp infection can reduce the diversity of intestinal microbes, resulting in the disturbance of intestinal microbiota and changes in the relative abundance of microbiota in children, while probiotic therapy can restore the diversity of intestinal microbes and intestinal microecological balance.

## 1 Introduction

*Helicobacter pylori* (Hp) is a gram-negative bacterium that is mainly found in the gastric mucosa. Hp is one of the common chronic infections in humans. Hp infection usually occurs in childhood and lasts until adulthood, affecting adult health. Once infected, it is difficult to be cleared naturally (Jiang, 2023). Globally, infection rates in developed and developing countries fluctuated between 8.9 and 72.8% (Segal et al., 2008). According to the epidemiological survey, the Hp infection rate of children in Europe, Asia, South America, and South Africa was 7.0–33.0%, 37.5–66.0%, 48.0–78.0%, and 87.0%, respectively (Goh et al., 2011). According to a study in 2021, the rate of Hp infection among children and adolescents in China was 20.55% (Zhou et al., 2023). The guidelines of the American Society of Hematology state that routine Hp testing and eradication treatment are not recommended for asymptomatic children infected with Hp (Neunert et al., 2011). However, Hp detection is recommended for children with gastrointestinal symptoms such as repeated abdominal discomfort, nausea, vomiting, belching, etc. And Hp eradication therapy can be considered for those with positive test results (Subspecialty Group of Gastroenterology, the Society of Pediatrics, Chinese Medical Association, National Children’s Medical Center Digestive Specialist Alliance, Editorial Board, Chinese Journal of Pediatrics, 2023). For Hp-positive children with functional gastrointestinal disorders, the eradication of Hp could lead to long-term relief of symptoms in some patients, and could be considered as a preferred treatment (Sugano et al., 2015). Therefore, Hp eradication therapy is recommended for children with Hp infection and functional abdominal pain.

Currently recognized first-line and second-line regimens to eradicate Hp infection in children require at least two antibiotics. These antibiotic therapy has many disadvantages (Seo et al., 2020) in the eradication of Hp infection in children, such as the limited choice of antibiotics in the eradication therapy, the increasing resistance rate of bacteria to antibiotics, and the low tolerance of children to adverse drug reactions (such as abdominal pain, diarrhea, nausea, vomiting, etc.). In recent years, the eradication of probiotics for Hp infection has gradually become a focal point in the research field. Probiotics have been proven to inhibit Hp infection, indicating their potential significance in combating this issue (Lv et al., 2015; Subspecialty Group of Gastroenterology, the Society of Pediatrics, Chinese Medical Association, National Children’s Medical Center Digestive Specialist Alliance, Editorial Board, Chinese Journal of Pediatrics, 2023). Compared to antibiotic therapy, probiotics rarely cause adverse reactions and do not increase strain resistance. Additionally, children have a higher tolerance and better compliance with probiotics. Therefore, the development of probiotic therapy for treating Hp infection in children is of great significance in academic research.

Studies have shown that probiotics can secrete antimicrobial substances, mainly including lactic acid, short-chain fatty acids, hydrogen peroxide, bactericin and so on. Lactic acid and short-chain fatty acids can reduce cytoplasmic pH and prevent the colonization of Hp, while hydrogen peroxide is associated with the destruction of pathogenic proteins, membrane lipids, and DNA of bacterial cells (Ji and Yang, 2020). In addition, probiotics can affect immune function and indirectly inhibit Hp infection (Sanders et al., 2019). *Clostridium butyricum* produces butyrate, which may exhibit bactericidal effects on Hp. *In vitro* studies utilizing the supernatant of butyrate culture and butyrate-producing bacteria have revealed their ability inhibit the growth of Hp, which is attributed to the destruction of the envelope of Hp cells (Yonezawa et al., 2012). And previous studies have proved that there is antagonistic interaction between *Clostridium butyricum* and Hp *in vitro* and *in vivo*, and the supernatant of *Clostridium butyricum* culture can inhibit the growth of Hp before and after pH adjustment (Takahashi et al., 2000), so it has a promising prospect in the treatment of Hp infection. In addition, supplementation of *Bacillus coagulans* has a positive effect on the function of the human immune system (Fu et al., 2021). It has been reported that the supplementation of *Bacillus coagulans* in the treatment of Hp infection with triple therapy can significantly improve the eradication rate and effectively reduce the incidence of adverse reactions (Wang, 2013). Currently, researches have demonstrated that the combination of *Clostridium butyricum* and *Bacillus coagulans* is capable of effectively eradicating Hp, with a higher eradication rate compared to either *Clostridium butyricum* or *Bacillus coagulans* alone (Zhang et al., 2020; Zhu, 2023). While there is compelling evidence suggesting that probiotic strains may not exert their effectiveness through colonization in the gastrointestinal tract, but rather by sharing genes and metabolites and directly influencing the intestinal barrier and immune cells (Wieërs et al., 2019), the precise mechanism of action of probiotics remains unclear. In particular, the mechanism of eradication of Hp by *Clostridium butyricum* and *Bacillus coagulans* is rarely reported. Based on this, this study intends to explore the potential impact of probiotic therapy on Hp infection in children through 16S rRNA gene sequencing technology and untargeted metabolomics.

## 2 Materials and methods

### 2.1 Study design

In this study, 24 children with Hp infection and functional abdominal pain were recruited from the pediatric clinic of Shanxi Bethune Hospital, Shanxi Province, China. Besides, 24 healthy children were recruited as a control group. The inclusion criteria were as follows: (1) Aged between 5 and 14 years old; (2) Hp infection confirmed by ^13^C urea breath test; (3) Functional abdominal pain meeting the diagnostic criteria of Rome IV, with no other organic diseases; (4) No previous Hp eradication therapy. The exclusion criteria included: (1) Children who do not follow medical advice resulting in invalid results; (2) Patients with a history of allergy to microecological preparations; (3) Individuals with a history of Hp eradication; (4) Those who have taken antibacterial drugs within the past month.

The administration regimen for children infected with Hp was as follows: *Clostridium butyricum* Powder, Live (Qingdao Eastsea Pharmaceutical Co., Ltd., lot No: B202301038), 1 bag (1.5 × 10^7^ CFU) per dose, 3 times a day; *Bacillus coagulans* Tablets, Live (Qingdao Eastsea Pharmaceutical Co., Ltd., lot No: S202302029), 3 tablets (5.25 × 10^7^ CFU) per dose, 3 times a day. Additionally, to alleviate functional abdominal pain in the included population as soon as possible, Lamb’s Tripe Extract and Vitamin B12 Granules (Xinjiang Biochemical Pharmaceutical Co., Ltd., lot No: 202210035, 1 bag per dose, 3 times a day) was added. The indications of this drug are upper abdominal discomfort, abdominal distention, loss of appetite caused by chronic gastritis, which is a protective agent for gastric mucosa with high safety and has a strong gastric acid buffer effect. The above three drugs were combined to treat Hp infected children for 4 weeks. And healthy children in the control group did not receive any interventions.

The gender, age, and weight data of each child were collected. After 4 weeks of probiotic medication, the subjects underwent the ^13^C urea breath test again at the gastroenterology department of Shanxi Bethune Hospital. We collected the Delta Over Baseline (DOB) value from two breath tests before and after medication, and determined whether Hp has been eradicated (DOB < 4.0 represents normal; DOB ≥ 4.0 represents Hp infection). In addition, gastrointestinal symptom rating scale (GSRS) scores were obtained before and 4 weeks after treatment. These measures were used to evaluate the antibacterial effect of probiotic therapy on Hp and the improvement of functional abdominal pain in the children, respectively. In addition, 10 mL sterile fecal sampling tubes were utilized to collect stool samples from healthy children and Hp infection children. For Hp infection children, the first stool samples were collected prior to treatment, the second after 2 weeks of treatment, and the third after 4 weeks of treatment. Following each collection, the samples were promptly stored in a refrigerator at −80°C for subsequent 16S rRNA gene sequencing analysis and untargeted metabolomic analysis.

This study was approved by the Ethics Review Committee of Shanxi Bethune Hospital (approval number: YXLL-2022-064), and all participants provided signed informed consent. Furthermore, this study has been registered in the Chinese Clinical Trial^1^ with the registration number ChiCTR2200062024.

### 2.2 16S rRNA gene sequencing analysis

The specific process for 16S rRNA gene sequencing analysis of children’s stool samples was as follows. Total genomic DNA was extracted from fecal samples using the M5635-02 OMEGA Soil DNA Kit (Omega Bio-Tek, Norcross, GA, United States). NanoDrop NC2000 spectrophotometer (Thermo Fisher Scientific, Waltham, MA, USA) and agarose gel electrophoresis were then used to measure the quantity and quality of the extracted DNAs. Then, forward primer 338F (5′-ACTCCTACGGGAGGCAGCA-3′) and reverse primer 806R (5′-GGACTACHVGGGTWTCTAAT-3′) were used in PCR amplification of the bacterial 16S rRNA gene V3-V4 region. The PCR amplification products were purified by VAHTSTM DNA Clean Beads (Nazyme, Nanjing, China) and quantified by the Quant-iT PicoGreen dsDNA Assay Kit (Invitrogen, Carlsbad, CA, USA). After the individual quantification step, amplicons were pooled in equal amounts, and pair-end 2 × 250 bp sequencing was performed using the Illlumina NovaSeq platform with NovaSeq 6,000 SP Reagent Kit (500 cycles) at Shanghai Metabo-Profile Biotechnology Co., Ltd. (Shanghai, China). Sequences were quality filtered, denoised, merged and chimera removed using the DADA2 plugin, then the effective sequences of each sample were obtained. USEARCH (version 10.0) was used to assign qualified sequences with similarity threshold exceeding 97% to an operation classification unit (OTU) for subsequent analysis.

Sequence data analysis were mainly performed using QIIME2 2019.4 (according to the official tutorials)^2^ and R packages (v3.2.0). Briefly, raw sequence data were demultiplexed using the demux plugin following by primers cutting with cutadapt plugin (Martin, 2011). Sequences were then quality filtered, denoised, merged and chimera removed using the DADA2 plugin (Callahan et al., 2016). Non-singleton amplicon sequence variants (ASVs) were aligned with mafft (Katoh et al., 2002) and used to construct a phylogeny with fasttree2 (Price et al., 2009). Alpha diversity metrics (Chao1, Shannon, Simpson), beta diversity metrics (weighted UniFrac, unweighted UniFrac) were estimated using the diversity plugin. Alpha diversity indices were visualized as box plots and betadiversity analysis was visualized via principal coordinate analysis (PCoA). Permutational multivariate analysis of variance (PERMANOVA) was used to compare bacterial abundance and diversity. Moreover, the online analysis platform Huttentower Lab Galaxy Huttentower was used for linear discriminant analysis (LEfSe).

### 2.3 Untargeted metabolomics analysis

The 20 mg freeze-dried fecal sample was transferred to a 1.5 mL centrifuge tube, followed by the addition of 1 mL of iced water for thorough mixing, and centrifugation for 15 min (13,000 rpm, 4°C) after ultrasonic processing for 20 min. The supernatant was then transferred. Then 1 mL of cold methanol (LC–MS grade, Thermo Fisher Scientific) was added to the remaining precipitate in the centrifuge tube and thoroughly mixed. After 20 min of ultrasonic processing, the mixture was centrifuged for 15 min (13,000 rpm, 4°C), and the supernatant was retained. The two kinds of supernatant obtained from the above two operations were, respectively, absorbed 500 μL and mixed, and then added 1 mL of cold acetonitrile (LC–MS grade, Thermo Fisher Scientific). After vortex mixing, the supernatant was retained after ultrasonic processing for 20 min and centrifugation for 15 min (13,000 rpm, 4°C). The supernatant was then evaporated to dryness using a high-speed vacuum concentrator. After drying, the product was redissolved in 100 μL 80% methanol solution and centrifuged for 15 min (13,000 rpm, 4°C) to obtain the supernatant for untargeted metabolomics analysis.

The LC–MS system for metabolomics analysis was composed of UltiMate 3,000 high performance liquid chromatography (Thermo Fisher Scientific, Waltham, MA, United States) and Thermo Scientific™ Q Exactive™ Hybrid Quadrupole-Orbitrap™ Mass Spectrometer (Thermo Fisher Scientific, Waltham, MA, USA). The liquid chromatography column used in the study was Waters Acquity UPLC HSS T3 column (1.8 μm, 2.1 mm × 100 mm) purchased from Waters, USA. The injection volume was 2 μL and the flow rate was 0.30 mL/min. The mobile phase consisted of 0.1% formic acid (LC–MS grade, Tokyo Chemical Industry) aqueous solution (A) and 0.1% formic acid-acetonitrile solution (B). The gradient elution conditions were as follows: 0 ~ 2 min, 98% A and 2% B; 3 ~ 5 min, 75% A and 25% B; 6 ~ 9 min, 50% A and 50% B; 10 ~ 12 min, 30% A and 70% B; 13 ~ 16 min, 15% A and 85% B; 17 ~ 18 min, 2% A and 98% B; 18.5 ~ 20 min, 98% A and 2% B. In this study, the ionization method of heated electrospray ionization (HESI) was utilized. The specific parameters were as follows: a spray voltage of 3,500 V (positive) /2500 V (negative); a capillary temperature of 320°C and a heater temperature of 300°C; the sheath gas flow rate was set at 35 arb and the auxiliary gas flow rate at 10 arb. The scanning mode was Full Scan/dd-MS2, with an m/z collection range of 100 ~ 1,500 in positive and negative ion switching collection mode. The resolution was MS Full Scan 35,000 FWHM and MS/MS 17500 FWHM. Additionally, the secondary fragmentation energies for MS were specified as 12.5 eV, 25 eV and 37.5 eV.

The original mass spectrometry data were processed using Compound Discover (version 3.3 SP2) software. Peak extraction, peak comparison, compound identification, and other data processing operations were conducted to obtain the peak table data of metabolites in the sample. Subsequently, the peak table data of metabolites were imported into SIMCA (version 14.1) software for further analysis. Principal component analysis (PCA) and orthogonal partial least squares discriminant analysis (OPLS-DA) were initially used to analyze the overall differences among the groups, among which OPLS-DA analysis also needs to use permutation test to verify the reliability of the model. Once the reliability of the OPLS-DA model was confirmed, group B-A, group B1-B, and group B2-B were analyzed separately by OPLS-DA to obtain variable importance in projection (VIP). By combining VIP values with Student-*t* test and fold change (FC) values, differential metabolites in group B, group B1, and group B2 were screened under specific conditions: VIP > 1, *p* < 0.05, FC > 1.5 or FC < 2/3. Finally, the differential metabolites were introduced into the online analysis platform of MetaboAnalyst 6.0^3^ for enrichment analysis of KEGG metabolic pathway.

### 2.4 Data processing and analysis

The sex ratio was analyzed using the Chi-square test in SPSS software, while other demographic data was analyzed using the Student’s *t* test. A significance level of *p* < 0.05 was employed to determine whether there were statistically significant differences between the data.

## 3 Results

### 3.1 Baseline characteristics of children

Stool samples of healthy children were categorized as group A (*n* = 24), while stool samples of children with Hp infection and functional abdominal pain were classified as group B (*n* = 24). After 2 weeks of treatment with probiotics, 15 children in group B submitted stool samples, which were recorded as group B1, numbered B1_1 ~ B1_15. And after 4 weeks of treatment with probiotics, 19 children in group B submitted stool samples, which were recorded as group B2, numbered B2_1 ~ B2_19. The demographic data of children in groups A and B, along with the DOB value, Hp eradication rate, and GSRS scores of children in group B before and after probiotic therapy are presented in Table 1. A total of 12 children with Hp infection underwent Hp reexamination after the completion of the four-week probiotic therapy period. Statistical analysis revealed a decreasing trend in the DOB value, and the Hp eradication rate was 33.33%. The decrease in DOB value meant that probiotic therapy may reduce the bacterial load of Hp, but the difference was not significant (*p* = 0.065) probably due to insufficient sample size. In addition, the eradication rate also proved that probiotic therapy had an inhibitory effect on Hp. Following the four-week probiotic therapy period, there was a significant reduction in GSRS scores (*p* < 0.001), indicating that the child’s gastrointestinal symptoms were reduced after medication. Therefore, it proved that probiotic therapy can effectively alleviate symptoms associated with functional abdominal pain among infected children.

**Table 1:** Baseline characteristics of children.

Name	A (*n* = 24)	B (*n* = 24)	B1 (*n* = 15)	B2 (*n* = 19)
Age (mean)	8.63	8.58	8.53	8.52
Gender ratio (male/female)	1.00	0.71	1.14	0.90
Weight (mean)	32.46	29.87	30.57	30.44
DOB value (mean)	/	39.50	/	16.36
Hp eradication rate	/	/	/	33.33%
GSRS score (mean)	/	5.68	/	1.68***

***p < 0.001.

### 3.2 Effect of probiotic therapy on gut microbiota

#### 3.2.1 Alpha diversity and beta diversity

**Figure 1:**
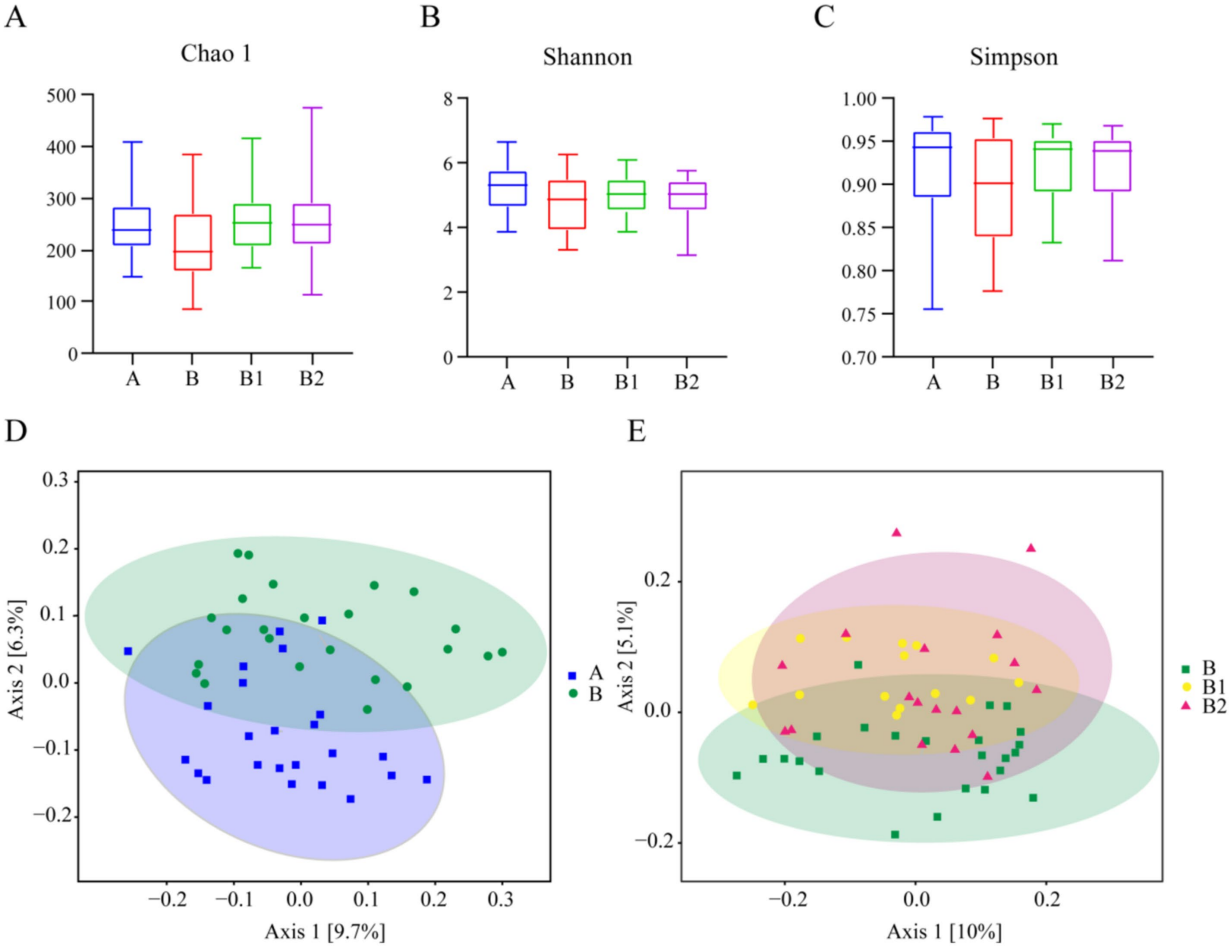
Alpha diversity and beta diversity. Alpha diversity of Group A, B, B1 and B2: (A) Chao1 index, (B) Shannon index, (C) Simpson index. Beta diversity of Group A and B: (D); beta diversity of Group B, B1 and B2: (E).

Alpha diversity is an index that can reflect the species richness and species diversity of samples. In this study, three alpha diversity indices (Chao 1, Shannon, Simpson) were chosen to assess the microbial community diversity in the stool samples, and the results are shown in Figure 1. The Chao 1 index was utilized to characterize species richness (Figure 1A), while the Shannon and Simpson indices were used to characterize species diversity (Figures 1B,C). It was found that there were no significant differences in the three indices among the four groups in this study.

In addition, beta diversity is utilized to assess the similarity of different samples in terms of species diversity. PCoA was employed in this study to analyze beta diversity among various groups, and the results are also presented in Figure 1. The closer the points on the coordinate diagram are, the higher the similarity between the samples is. The findings revealed no significant difference in beta diversity between healthy children and Hp-infected children, and probiotic therapy did not result in a significant alteration in the diversity of gut microbes among children after 2 and 4 weeks of treatment.

#### 3.2.2 Gut microbiota composition

**Figure 2:**
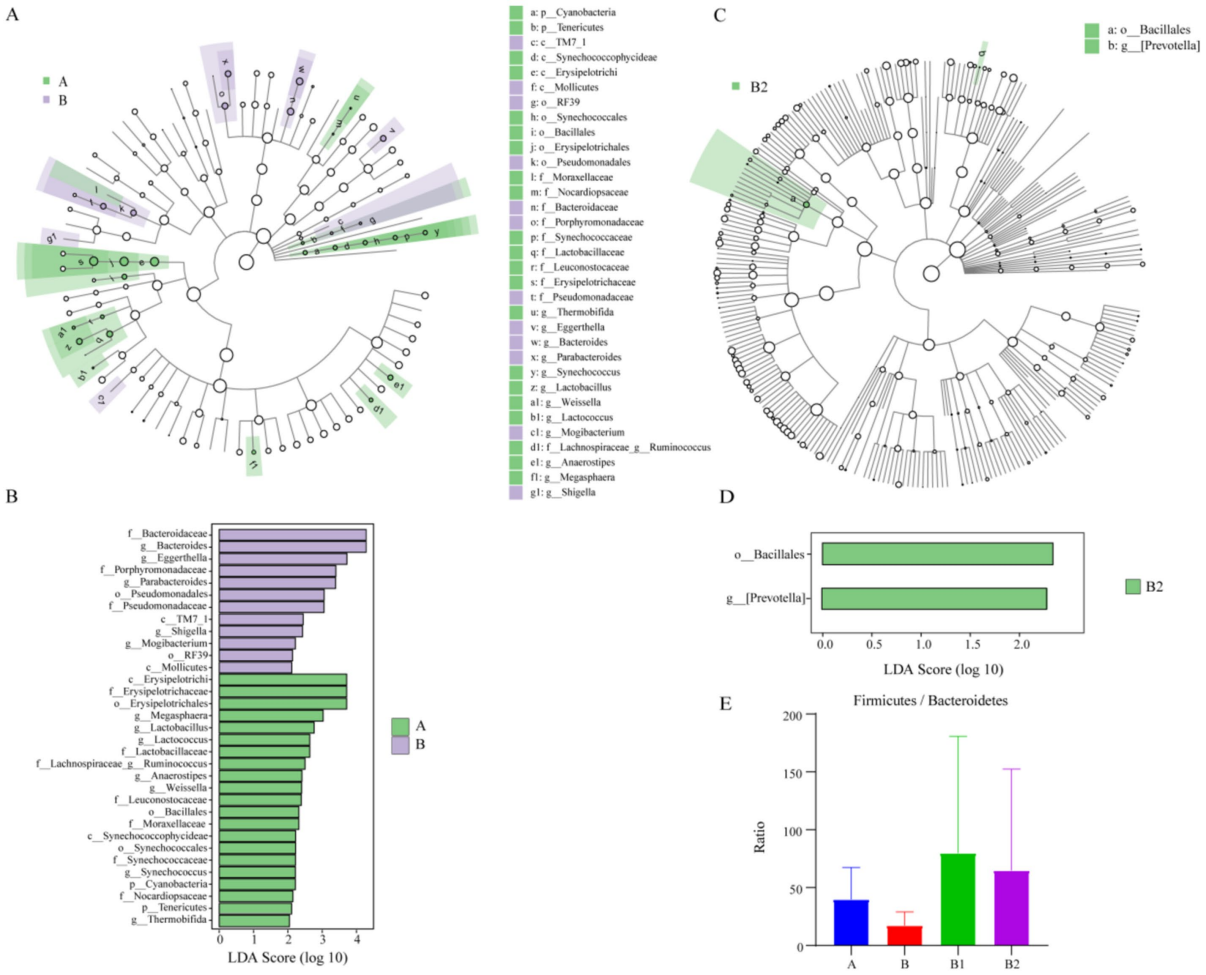
Results of LEfSe analysis and F/B value. The enriched gut microbiota in group A and group B: (A,B); the enriched gut microbiota in group B2: (C,D). Ratio of *Firmicutes* to *Bacteroidetes* among different groups: (E).

LEfSe analysis is commonly utilized to identify the enriched gut microbiota within each group. In this study, LDA > 2.0 was used as the criterion (Segata et al., 2011) to screen the enriched gut microbiota from phylum to genus level between group A and group B at first. The aim was to identify variations in gut microbiota between healthy children and those infected with Hp. The results are presented in Figure 2. The characteristic gut microbiota species enriched in group A and group B were 21 and 12, respectively. In Group A, the enriched gut microbiota included *Erysipelotrichi*, *Erysipelotrichaceae*, *Lactobacillus*, *Lactococcus*, *Lactobacillaceae*, etc. Meanwhile, the enriched gut microbiota in group B were *Bacteroides*, *Eggerthella*, *Porphyromonadaceae*, *Parabacteroides*, *Pseudomonadales*, etc. Besides, using LDA > 2.0 as the criterion, this study conducted LEfSe analysis to compare the differences in gut microbiota composition from phylum to genus level among groups B, B1 and B2, in order to identify characteristic microorganisms enriched in the gut of children infected with Hp before treatment, 2 weeks after probiotic treatment, and 4 weeks after probiotic treatment. The results are presented in Figure 2, showing that only two characteristic microorganisms, *Bacillales* and *Prevotella*, were found in group B2.

The ratio of the relative content of *Firmicutes* and *Bacteroidetes* (F/B value) is commonly utilized to assess the health of the intestinal microecological environment (Wei et al., 2021). Therefore, the F/B value of each group was analyzed in this study, and the results indicated that Hp infection was associated with a decrease in F/B value, while probiotic therapy was associated with an increase in F/B value, as depicted in Figure 2. However, possibly due to the small sample size, these differences did not show statistical significance.

**Figure 3:**
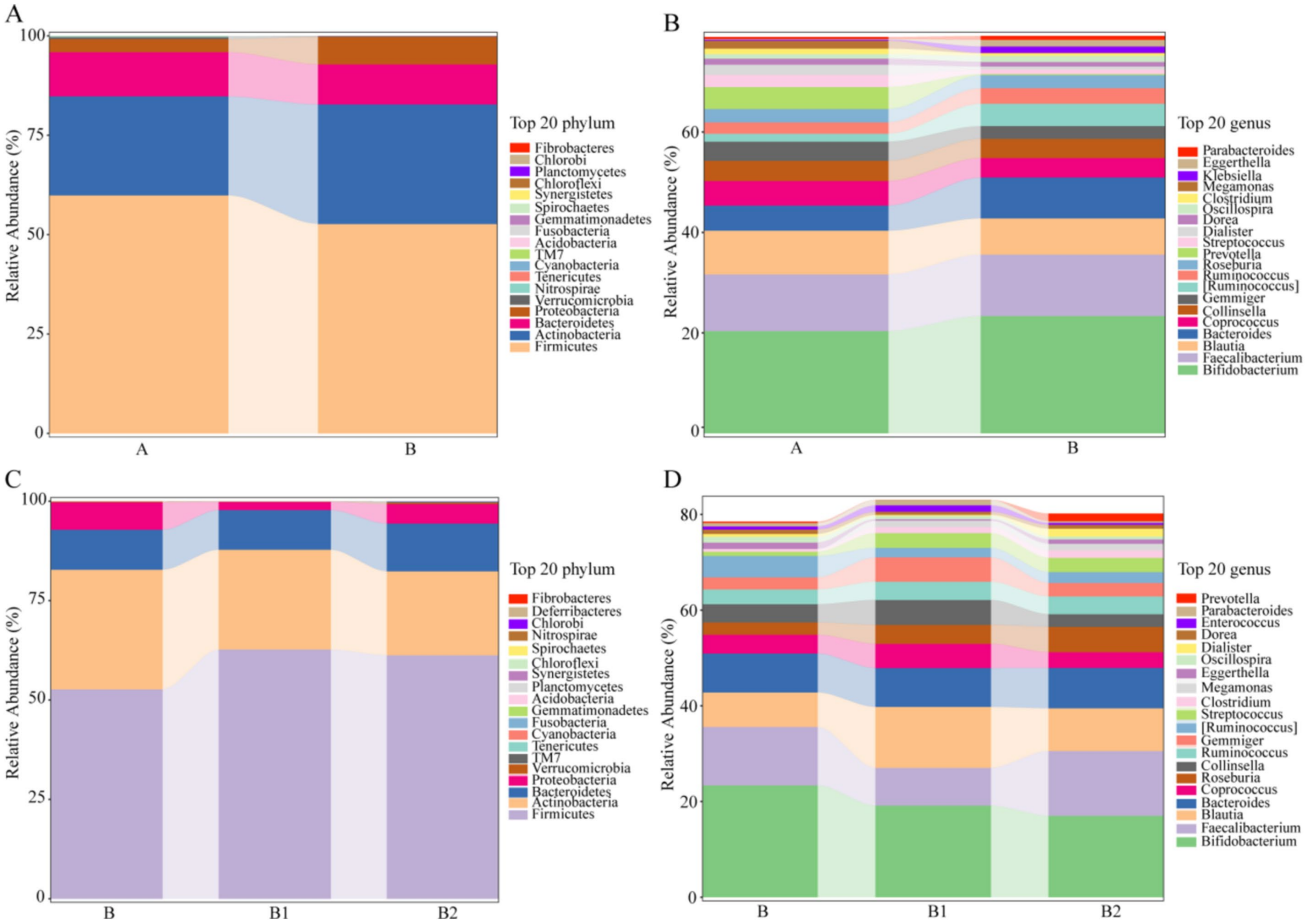
The relative abundance of the top 20 gut microbiota in different groups at the phylum/genus level. (A,B) The relative abundance of the top 20 gut microbiota in group A and group B at the phylum/genus level. (C,D) The relative abundance of the top 20 gut microbiota in group B, group B1 and group B2 at the phylum/genus level.

In order to comprehensively evaluate the effects of probiotic therapy on the microbiota composition of children infected with Hp, this study also conducted statistical analysis on the top 20 gut microbiota with relative abundance at phylum level and genus level, and the results are shown in Figure 3. At phylum level and genus level, with FC > 1.5 or FC < 2/3 as the criterion, the gut microbiota with recovery trends in relative content in group B1 and B2 were determined, respectively. As displayed in Table 2, at the phylum level, there were each 5 gut microbiota with recovery trends in group B1 and group B2. Meanwhile, at the genus level, 6 gut microbiota in group B1 and 7 gut microbiota in group B2 were found to have recovery trends, and the abundance of *Parabacteroides* decreased significantly in group B2 (*p* = 0.0465).

**Table 2:** The gut microbiota which had trends of recovery in the content after probiotic treatment.

No.	Name	FC (B/A)	FC (B1/B)	FC (B2/B)
1	*p_Proteobacteria*	2.1068 ↑	0.2990 ↓	/
2	*p_Verrucomicrobia*	0.3510 ↓	/	1.7743 ↑
3	*p*_*TM7*	14.6923 ↑	0.3500 ↓	/
4	*p_Acidobacteria*	1.6875 ↑	0.2540 ↓	0.3457 ↓
5	*p_Fusobacteria*	0.3333 ↓	15.4286 ↑	4.7407 ↑
6	*p_Gemmatimonadetes*	4.3333 ↑	0.1978 ↓	0.6154 ↓
7	*p_Planctomycetes*	0.6000 ↓	/	8.4444 ↑
8	*g_Gemmiger*	0.6649 ↓	2.0055 ↑	/
9	*g_Ruminococcus*	2.8416 ↑	0.4497 ↓	0.5100 ↓
10	*g_Prevotella*	0.0518 ↓	/	7.0075 ↑
11	*g_Streptococcus*	0.3777 ↓	3.3858 ↑	3.2730 ↑
12	*g_Dialister*	0.2909 ↓	/	2.8376 ↑
13	*g_Clostridium*	0.4870 ↓	2.2998 ↑	2.9022 ↑
14	*g_Megamonas*	0.0303 ↓	28.2413 ↑	29.1605 ↑
15	*g_Eggerthella*	9.5172 ↑	0.2341 ↓	/
16	*g_Parabacteroides*	1.9146 ↑	/	0.4689 ↓*

In the table, “↑” represents up-regulation of the gut microbiota content; “↓” means down-regulation of the gut microbiota content; and “*” means *p* < 0.05.

### 3.3 Effect of probiotic therapy on metabolites

#### 3.3.1 Variety of fecal metabolic profiles

**Figure 4:**
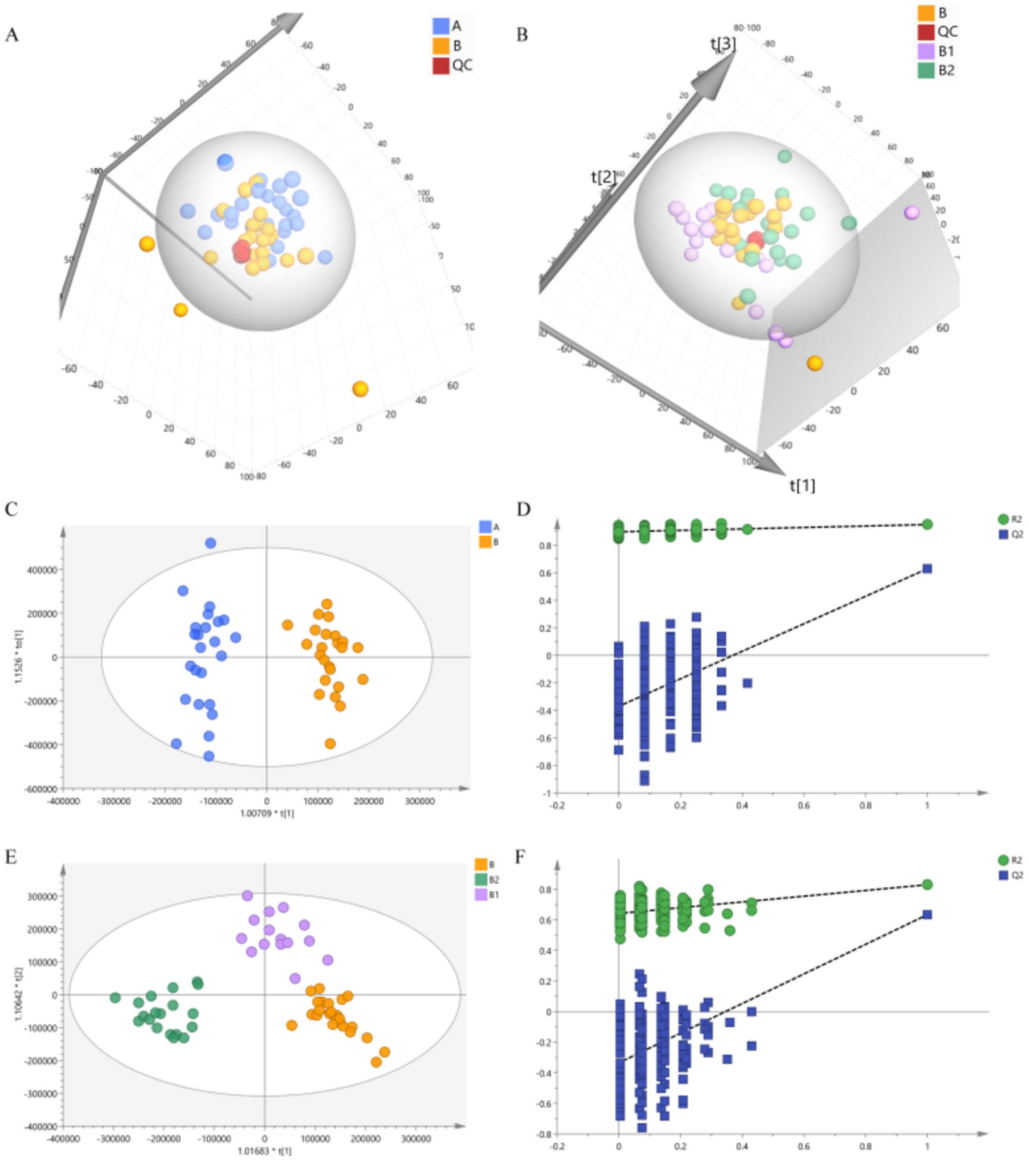
Results of PCA and OPLS-DA. (A) PCA result of group A and group B. (B) PCA result of group B, group B1 and group B2. (C,D) Results of OPLS-DA and permutations test of group A and group B. (E,F) Results of OPLS-DA and permutations test of group B, group B1 and group B2.

Unsupervised PCA analysis can effectively capture the overall differences between various sample groups and the degree of variation between samples within the group. In this study, PCA analysis was performed on group A and group B, as well as on group B, group B1 and group B2, in order to examine the disparities in metabolic profiles between Hp infected children and healthy children, as well as the differences among Hp infected children before medication and at 2 and 4 weeks post-medication. The 3D score charts of PCA are depicted in Figure 4, demonstrating discernible separation trends between group A and group B, as well as among group B, group B1, and group B2. These observation suggested that there were differences in the metabolite profiles between Hp infected children and healthy children, and Hp infected children exhibit changes in their metabolite profiles following probiotic therapy. Additionally, the tightly clustered QC samples indicated that the analysis method is stable and exhibits good repeatability.

To further analyze the differences between the groups, this study utilized a supervised OPLS-DA analysis method. In comparison to PCA analysis, OPLS-DA analysis is more sensitive to inter-group differences and can achieve better separation results (Thévenot et al., 2015). The OPLS-DA analysis results are presented in Figure 4, clear separation were observed among the groups, which indicate significant differences in the metabolite profiles between each group. Additionally, the permutations test was conducted to verify the reliability of the OPLS-DA model. The verification results depicted in Figure 4 showed that Q^2^ intersected with the vertical axis on the negative half axis, confirming the reliability of the OPLS-DA model.

#### 3.3.2 Screening of differential metabolites

OPLS-DA was employed to analyze group B and group A, and VIP values were obtained. In this study, these values were combined with the Student’s *t-*test and FC value to screen for differential metabolites between the groups. The screening conditions were set as VIP > 1, *p* < 0.05, FC > 1.5 or FC < 2/3, and the resulting differential metabolites are presented in Table 3. Group B exhibited 50 differential metabolites, indicating that Hp infection may lead to significant changes in these metabolites.

**Table 3:** Differential metabolite information of group B, B1, and B2.

No.	Name	Chemical formula	Class	B/A	B1/B	B2/B
1	Uracil	C_4_H_4_N_2_O_2_	Pyrimidones	* ↓	/	/
2	D-(+)-Proline	C_5_H_9_NO_2_	Proline and derivatives	** ↑	/	/
3	2-Hydroxyvaleric acid	C_5_H_10_O_3_	Hydroxy fatty acids	***↑	/	/
4	Thymine	C_5_H_6_N_2_O_2_	Hydroxypyrimidines	***↑	/	/
5	4-Oxoproline	C_5_H_6_NO_3_	Proline and derivatives	* ↑	/	/
6	Mesaconic acid	C_5_H_6_O_4_	Methyl-branched fatty acids	***↑	* ↓	* ↓
7	D-(+)-Pipecolinic acid	C_6_H_11_NO_2_	Alpha amino acids	* ↑	* ↓	/
8	6-Hydroxycaproic acid	C_6_H_12_O_3_	Medium-chain hydroxy acids and derivatives	***↑	/	/
9	Hypoxanthine	C_5_H_4_N_4_O	Hypoxanthines	* ↓	/	* ↑
10	D-α-Hydroxyglutaric acid	C_5_H_8_O_5_	Short-chain hydroxy acids and derivatives	***↑	** ↓	** ↓
11	DL-Glutamine	C_5_H_10_N_2_O_3_	L-alpha-amino acids	***↓	** ↑	** ↑
12	Guanine	C_5_H_5_N_5_O	Purines and purine derivatives	* ↓	* ↑	* ↑
13	Xanthine	C_5_H_4_N_4_O_2_	Xanthines	** ↑	/	/
14	N-Acetylhistamine	C_7_H_11_N_3_O	N-acetyl-2-arylethylamines	* ↑	/	/
15	Daminozide	C_6_H_12_N_2_O_3_	Straight chain fatty acids	* ↑	** ↓	/
16	7-Methylguanine	C_6_H_7_N_5_O	Hypoxanthines	* ↑	/	/
17	N-Acetyl-L-leucine	C_8_H_15_NO_3_	Leucine and derivatives	***↑	/	/
18	4-Hydroxyphenyllactic acid	C_9_H_10_O_4_	Phenylpropanoic acids	* ↑	* ↓	** ↓
19	apronalide	C_9_H_16_N_2_O_2_	N-acyl ureas	** ↑	/	/
20	Pivagabine	C_9_H_17_NO_3_	Gamma amino acids and derivatives	** ↑	/	/
21	Alanylproline	C_8_H_14_N_2_O_3_	Dipeptides	* ↑	/	/
22	N-Acetyl-DL-glutamic acid	C_7_H_11_NO_5_	Glutamic acid and derivatives	* ↑	/	/
23	Glycylleucine	C_8_H_16_N_2_O_3_	Dipeptides	** ↑	/	/
24	Capryloylglycine	C_10_H_19_NO_3_	N-acyl-alpha amino acids	** ↑	/	/
25	N-acetyl-L-2-aminoadipic acid	C_8_H_13_NO_5_	N-acyl-alpha amino acids	* ↑	* ↓	/
26	3-O-ETHYL ASCORBIC ACID	C_8_H_12_O_6_	Sugar Acids	** ↑	/	/
27	Valylvaline	C_10_H_18_N_2_O_3_	Dipeptides	* ↓	/	* ↑
28	propionylcarnitine	C_10_H_19_NO_4_	Acyl carnitines	* ↑	/	/
29	Uridine	C_9_H_12_N_2_O_6_	Pyrimidine nucleosides	* ↓	/	/
30	L-gamma-Glutamyl-L-valine	C_10_H_18_N_2_O_5_	Dipeptides	** ↑	/	/
No.	Name	Chemical formula	Class	B/A	B1/B	B2/B
31	N2-(D-1-Carboxyethyl)-L-arginine	C_9_H_18_N_4_O_4_	Amino Acids	* ↑	/	/
32	2’-Deoxyinosine	C_10_H_12_N_4_O_4_	Purine 2′-deoxyribonucleosides	** ↓	/	/
33	Tyrosylalanine	C_12_H_16_N_2_O_4_	Dipeptides	** ↑	/	/
34	Homovanillic acid sulfate	C_9_H_10_O_7_S	Phenylsulfates	* ↓	/	/
35	Arabinosylhypoxanthine	C_10_H_12_N_4_O_5_	Purine nucleosides	* ↓	/	/
36	Adenosine	C_10_H_13_N_5_O_4_	Purine nucleosides	** ↓	/	/
37	Genistein	C_15_H_10_O_5_	Isoflavones	* ↑	/	/
38	Musk ambrette	C_12_H_16_N_2_O_5_	Dinitrotoluenes	** ↑	** ↓	/
39	Palmitic Acid	C_16_H_32_O_2_	Long-chain fatty acids	***↑	***↓	***↓
40	8-hydroxy-deoxyguanosine	C_10_H_13_N_5_O_5_	Purine 2′-deoxyribonucleosides	* ↓		/
41	3-Hydroxy-palmitic acid methyl ester	C_17_H_34_O_3_	fatty acid methyl ester	* ↑	/	/
42	2-Deoxy-2,3-dehydro-n-acetyl-neuraminic acid	C_11_H_17_NO_8_	Acetamides	* ↑	/	/
43	(Ac)2-L-Lys-D-Ala	C_13_H_23_N_3_O_5_	Dipeptides	* ↑	* ↓	/
44	3,4,3′-Tri-O-methylellagic acid	C_17_H_12_O_8_	Benzopyrans	* ↑	/	/
45	Docosahexaenoic acid ethyl ester	C_24_H_36_O_2_	Fatty acid esters	* ↑	/	/
46	Leucyl-leucyl-norleucine	C_18_H_35_N_3_O_4_	Peptide	***↑	/	/
47	Androsterone sulfate	C_19_H_30_O_5_S	Sulfated steroids	* ↑	/	/
48	LysoPE(16:0/0:0)	C_21_H_44_NO_7_P	Lipids and lipid-like molecules	* ↓	/	/
49	Codonocarpine	C_26_H_31_N_3_O_5_	Indole Alkaloids	***↑	/	/
50	Cer(d18:0/14:0)	C_32_H_65_NO_3_	Long-chain ceramides	** ↑	/	/

In the table, “↑” represents up-regulation of content of metabolites; and “↓” means down-regulation of content of metabolites (“*” mean *P* < 0.05; “**” mean *p* < 0.01; and “***” mean *P* < 0.001).

Subsequently, further analyses were conducted on the metabolites of group B1 and group B, as well as group B2 and group B. Similarly, using VIP > 1, *p* < 0.05, FC > 1.5 or FC < 2/3 as criteria, metabolites with opposite adjustment trends compared to those in group B were screened for group B1 and group B2. In the end, there were 11 metabolites exhibiting opposite adjustment trends in the B1 group and 8 metabolites showing opposite adjustment trends in the B2 group. These metabolites were closely associated with the eradication of Hp through probiotic therapy.

#### 3.3.3 Analysis of metabolic pathway

**Figure 5:**
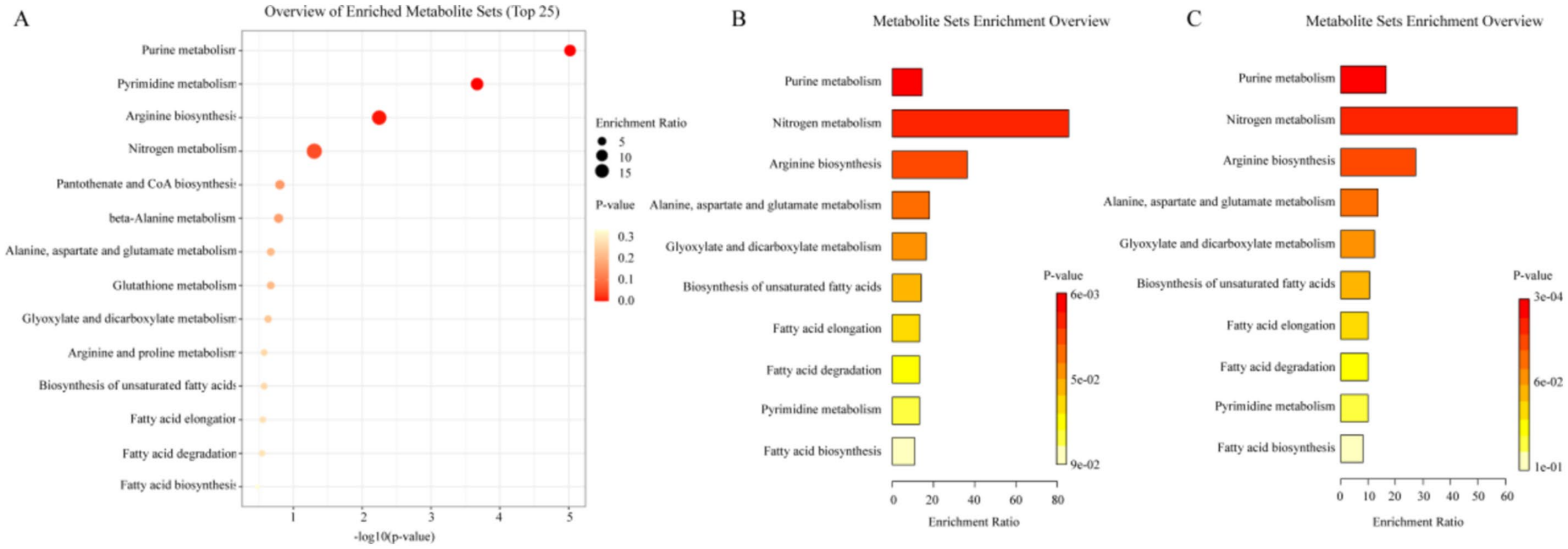
Analysis results of metabolic pathway. (A) Metabolic pathways of differential metabolites of group B. (B) Metabolic pathways of differential metabolites of group B1. (C) Metabolic pathways of differential metabolites of group B2.

Firstly, the differential metabolites of group B were introduced into the online analysis platform, MetaboAnalyst 6.0, for enrichment analysis of KEGG metabolic pathway, and the results are shown in Figure 5. Hp infection in children was closely related to 14 metabolic pathways including purine metabolism, pyrimidine metabolism, arginine biosynthesis, nitrogen metabolism, pantothenate and CoA biosynthesis, beta-alanine metabolism, alanine, aspartate and glutamate metabolism, glutathione metabolism, glyoxylate and dicarboxylate metabolism, arginine and proline metabolism, biosynthesis of unsaturated fatty acids, fatty acid elongation, fatty acid degradation, and fatty acid biosynthesis.

Subsequently, the metabolites in group B1 and group B2 with regulatory trends opposite to those in group B were also incorporated into the analysis platform for KEGG metabolic pathway enrichment analysis. The results are presented in Figure 5. These metabolites in both B1 and B2 group encompassed 10 metabolic pathways, namely purine metabolism, nitrogen metabolism, arginine biosynthesis, alanine, aspartate and glutamate metabolism, glyoxylate and dicarboxylate metabolism, biosynthesis of unsaturated fatty acids, fatty acid elongation, fatty acid degradation, pyrimidine metabolism, fatty acid biosynthesis. This suggested that these 10 metabolic pathways might be the key pathways for the efficacy of probiotic therapy, and the prolonged duration of medication (2 weeks/4 weeks) did not produce additional changes in metabolic pathways.

## 4 Discussion

### 4.1 Impact of probiotic therapy on gut bacteria composition

After 4 weeks of probiotic therapy, the DOB value of Hp infected children showed a decreasing trend, and the Hp eradication rate was 33.33%, suggesting that probiotic therapy could inhibit Hp to a certain extent. In addition, the GSRS scores were significantly reduced after 4 weeks of probiotic therapy, indicating that probiotic therapy can significantly alleviate functional abdominal pain symptoms in children with Hp infection.

Although Hp infection did not significantly alter alpha diversity of the gut microbiota in children, the alpha diversity exhibited a decrease tendency, which was in line with previous studies reporting an inverse relationship between microbial diversity and Hp abundance (Das et al., 2017). There was no significant difference in beta diversity between healthy children and those infected with Hp, and probiotic therapy failed to significantly alter the beta diversity of the gut microbiota in children after 2 and 4 weeks of treatment. Whether probiotics can significantly alter gut microbiota diversity is still a controversial issue (Kristensen et al., 2016). Most randomized controlled trials indicated that there were no significant differences in alpha diversity and beta diversity between the probiotic group and the placebo group (Chen et al., 2021). But some studies have also reported contrasting findings, indicating that certain probiotics can significantly influence alpha and beta diversity during intervention periods of 90 days (Rahayu et al., 2021) and 4 weeks (Ferrario et al., 2014), respectively. Interindividual variations, duration of treatment, smaller sample sizes, diverse evaluation tools, and various types of probiotics may account for the inconsistent results observed. The F/B ratio, a commonly employed metric to reflect the status of gut microbial ecosystem health, showed a downward trend in children with Hp infection in this study. Furthermore, the intestinal tracts of children infected with Hp demonstrated a notable enrichment of microorganisms implicated in gut dysbiosis, such as *Porphyromonadaceae* and *Shigella* (Laffin et al., 2019). And probiotic therapy was related to the recovery of F/B values. The enriched *Bacillales* in the probiotic treatment group was also one of the enriched species in the gut of healthy children. In Hp-infected children, an increasing trend in the abundance of *Proteobacteria* was observed, whereas a corresponding decrease in the abundance of *Streptococcus* was noted. These outcomes were congruent with the findings reported in other relevant studies (Klymiuk et al., 2017; Schulz et al., 2018; He et al., 2019). Following probiotic therapy, the abundances of both microbiota were modulated toward their original levels. Previous studies have reported that probiotic supplementation can enhance the abundance of *Prevotella* (Rahayu et al., 2021). The enrichment of *Prevotella* in the probiotic-treated group observed in this study further validated this notion. Moreover, probiotic therapy significantly reduced the abundance of *Parabacteroides*, downregulated the levels of *Ruminococcus*, *Eggerthella* and *TM7*, while upregulating the abundances of *Planctomycetes*, *Gemmiger*, *Dialister*, and others. The aforementioned results suggested that Hp infection could lead to an imbalance in gut microbiota, causing alterations in microbial community structure. Conversely, probiotic therapy could modulate the microbial community structure, restore intestinal microecological balance and exert an inhibitory effect on Hp.

The abundance of *Verrucomicrobia* is closely associated with intestinal health, as it has the capability to produce short chain fatty acids (SCFAs) such as propionic acid and butyric acid. These SCFAs play a crucial role in maintaining intestinal health and regulating the immune system (Schlesner, 2006). Furthermore, like *Verrucomicrobia*, *Clostridium*, *Fusobacteria*, and *Megamonas* were also identified as producers of SCFAs like butyrate (Brennan and Garrett, 2019; Martín-Núñez et al., 2019; Cao et al., 2022), but their abundance in HP-infected children were lower than in healthy children. Hp can overcome the epithelial barrier and further destroy the tight mucosal defense barrier (Marques et al., 2021), eventually triggering chronic inflammation. Butyrate, which is the primary SCFA produced through bacterial fermentation of dietary fiber, can enhance mucosal homeostasis by exerting beneficial effects on innate and adaptive immune cells and epithelial barrier function (Cushing et al., 2015). Previous studies have demonstrated that butyrate may exhibit a bactericidal effect on Hp. Researchers utilized butyrate and supernatant of butyrate-producing bacteria for *in vitro* research, and the results showed that both of them had inhibitory effects on the growth of Hp, and potentially destructive abilities on Hp biofilm (Yonezawa et al., 2012). Compared with healthy children, the abundance of these butyrate-producing enterobacteria decreased in Hp-infected children, but increased after probiotic treatment, suggesting that probiotic therapy can adjust the abundance of these butyrate-producing enterobacteria to inhibit Hp.

N-nitroso compounds are often considered as carcinogenic factors, which are related to chronic bacterial infection. The risk of Hp-related gastric cancer was related to the increase of nitrite in the stomach and the formation of N-nitroso compounds (Leach et al., 1987). Recent studies showed that nitrate and nitrite were biological activity reservoirs of nitric oxide (NO), and NO might play an important regulatory role in human body, but this biological activation required the existence of symbiotic bacteria, because mammals lacked specific reductase (Lundberg et al., 2008). Moreover, some studies showed that acidified nitrite had bactericidal effect on Hp (Dykhuizen et al., 1998). Therefore, nitrate-nitrite-NO pathway might play an important regulatory mechanism in people infected with Hp (Chen et al., 2018). *Gemmatimonadetes* might be involved in the nitrogen cycle, which could use a variety of organic and inorganic substances as carbon and energy sources, including nitrate (Oshiki et al., 2022). Additionally, *Acidobacteria* could also play a role in nitrogen cycle, as previous studies have demonstrated its ability to utilize nitrite as a nitrogen source (Kielak et al., 2016). In this study, the abundance of the above two kinds of gut microbiota related to nitrate-nitrite-NO pathway were up-regulated in children infected with Hp, but down-regulated in children treated with probiotics, suggesting that probiotic therapy may inhibit Hp by regulating such intestinal bacteria.

### 4.2 Impact of probiotic therapy on metabolic profile

Purine and pyrimidine are typically utilized for the synthesis of nucleotides, which serve as precursors of nucleic acids and significant participants in diverse biological processes (Marais et al., 1999). According to studies, numerous microorganisms, including infectious pathogens, lacked the pathways for *de novo* purine biosynthesis. Consequently, these microorganisms could only retrieve free purine and purine nucleotides from the environment to fulfill their purine requirements, a phenomenon known as the salvage pathways (Ullman and Carter, 1995; El Kouni, 2003). In recent years, it has been discovered that Hp also falls into this category of microorganisms lacking the pathways for *de novo* purine biosynthesis, yet it possessed potent salvage pathways of purine (Liechti and Goldberg, 2012). *In vitro* studies have demonstrated that substances targeting the purine uptake and processing mechanism of Hp have potential antibacterial effects (Gollapalli et al., 2010; Hedstrom et al., 2011). Furthermore, a previous study which using bismuth to inhibit Hp showed significant adjustments in the metabolic pathways of purine and pyrimidine in Hp positive patients after treatment (Yao et al., 2021), which was consistent with the findings of this study. The results of KEGG metabolic pathway analysis of differential metabolites in children infected with Hp indicated that Hp infection could impact purine and pyrimidine metabolism in children. Additionally, the KEGG metabolic pathway analysis of significantly reversed metabolites in the probiotic treatment group also implicated purine and pyrimidine metabolism, suggesting that probiotic therapy may influence nucleotide metabolism of Hp by regulating these pathways to inhibit its growth.

Ammonia serves as the primary nitrogen source for Hp, and it has demonstrated the existence of a nitrogen-or ammonia-dependent regulatory system in Hp metabolism (Mobley et al., 2001). Hp facilitates the generation of reactive nitrogen species (RNS) in the human stomach, which have been implicated in Hp-associated inflammation as well as DNA damage (Shimizu et al., 2017). Macrophages can utilize L-arginine as a substrate to produce NO via the induction of inducible nitric oxide synthase (Tsuji et al., 1996). Furthermore, *in vitro* experiments have demonstrated the bactericidal effect of NO against Hp (Kuwahara et al., 2000). Relativistically, Hp can also inhibit the production of NO by inducing macrophage apoptosis and synthesizing arginase, among other mechanisms (Gobert et al., 2001; Bussière et al., 2005). In this study, nitrogen metabolism pathway was enriched in Hp-infected children and was also one of the enriched pathways in children after probiotic treatment. This phenomenon may be attributed to the aforementioned interplay between NO and Hp, suggesting that probiotic therapy potentially inhibit Hp by modulating nitrogen metabolism in the human body.

Studies have demonstrated that amino acids not only serve as energy and nitrogen sources for Hp, but also play a pivotal role in protein synthesis and bacterial colonization, and they can determine the virulence and stress resistance of Hp strains (Leduc et al., 2010). Substances with bactericidal effects against Hp strains, such as bismuth agents, can lead to a decrease in amino acid abundance within the Hp growth environment, thereby inhibiting the growth of Hp strains (Han et al., 2018). Furthermore, it has been reported in other study that following the use of Hp eradication medications, the downregulated amino acid metabolic pathways in Hp include the biosynthesis of phenylalanine, tyrosine, tryptophan, and lysine, as well as the metabolism of alanine, aspartic acid, glutamic acid, arginine, and proline (Yao et al., 2021). In the present study, probiotic therapy primarily modulated the arginine biosynthesis pathway and the metabolism of alanine, aspartic acid, and glutamic acid, suggesting that these two pathways may represent potential mechanisms underlying the antibacterial effects of probiotics.

The probiotic therapy utilized in this study included *Clostridium butyricum*, which can increase the abundance of SCFAs in the body (Okamoto et al., 2000). SCFAs, including acetate, propionate, butyrate and valeric acid, play a crucial role in maintaining the normal biological activity of the host (Agus et al., 2021). Besides, SCFAs serve as one of the primary antibacterial substances secreted by probiotics and have various functions such as preserving intestinal barrier integrity (Dalile et al., 2019), promoting the induction and expansion of intestinal regulatory T cells and macrophages (Kim, 2021; Traxinger et al., 2022), combating cancer and oxidation (Liu et al., 2021), inhibiting inflammation induced by pathogens (He et al., 2020), among others. However, it was noteworthy that SCFAs was not prominently featured among the differential metabolites identified by LC–MS in this study due to its strong volatility (He et al., 2020). Additionally, it has been reported that palmitic acid, a long-chain fatty acid, can impact the virulence of Hp strains and regulate gene expression (Valdez-Salazar et al., 2021), thereby enhancing the motility and adhesion of Hp. The results of this study showed a significant increase in palmitic acid levels in children infected with Hp (*p* < 0.001), but a significant decrease in the probiotic treatment group (*p* < 0.001). This suggested that probiotic therapy may influence the motility and adhesion of Hp by regulating the content of palmitic acid. Some studies have suggested that certain fatty acids, particularly polyunsaturated fatty acids, exhibited a bactericidal effect on Hp, and the efficacy of their bactericidal activity would increase with the degree of unsaturation (Sun et al., 2003). In addition, it has been found that certain fatty acids could inhibit the growth of Hp in a dose-dependent manner and induce the transformation of bacteria from bacillus to coccus, leading to a decrease in bacterial activity (Correia et al., 2012). In summary, fatty acids play a significant role in inhibiting Hp, and probiotic therapy in this study may exert an antibacterial effect by regulating metabolic pathways related to fatty acids *in vivo*.

Glyoxylic acid and dicarboxylic acid are crucial metabolites in organisms, playing a role in energy metabolism. The tricarboxylic acid cycle is the primary source of cell energy (Duggleby and Jackson, 2002), and the metabolism of glyoxylic acid and dicarboxylic acid can regulate this cycle (Kang et al., 2021). This study suggested that Hp infection may impact the metabolism of glyoxylic acid and dicarboxylic acid in children, potentially affecting the energy metabolism pathway. Furthermore, an experiment involving bismuth eradication of Hp indicated that bismuth can affect various metabolic pathways, including glyoxylic acid or dicarboxylic acid metabolism, suggesting a relationship between this metabolic pathway and bismuth’s inhibition of Hp growth (Yao et al., 2021). In conclusion, regulating the metabolic pathways of glyoxylic acid and dicarboxylic acid may be a potential mechanism for probiotic therapy to inhibit Hp.

In this study, healthy children and Hp infected children were recruited, and probiotic therapy was provided for Hp infected children for 4 weeks. Fecal samples were collected from healthy children, as well as from Hp infected children before and after probiotic therapy. Subsequently, 16S rRNA gene sequencing analysis and untargeted metabonomics analysis were conducted on the aforementioned samples to investigate the potential mechanism of probiotic therapy in eradicating Hp. Although the eradication rate of probiotic therapy currently lags behind that of antibiotics, the emergence of this therapy offers hope to individuals who are unable to tolerate conventional treatment. This study thoroughly analyzes the potential mechanism of probiotic therapy in eradicating Hp, providing a valuable reference for identifying potential targets and developing new drugs to combat Hp. This study also has several limitations. Firstly, due to the cost limitation, the sample size in this study is not large, and it needs to be expanded for further research in the future. Secondly, this study found that probiotic therapy can affect the abundance of some intestinal microorganisms that can produce SCFAs, but SCFAs was not found in the results of untargeted metabonomics based on LC–MS, which was caused by the volatile characteristics of SCFAs, and it needs to be verified by targeted metabonomics based on GC–MS in the future.

## 5 Conclusion

In conclusion, this study demonstrated that probiotic therapy can help maintain intestinal microecological balance, regulate the abundance of butyrate-producing bacteria and intestinal microbiota related to the nitrate-nitrite-NO pathway. Furthermore, probiotic therapy was found to regulate pyrimidine and purine metabolic pathways, nitrogen metabolic pathways, amino acid-related metabolic pathways, fatty acid-related metabolic pathways, as well as glyoxylic acid and dicarboxylic acid metabolic pathways in children. The aforementioned approaches might be potential mechanisms of probiotic therapy for Hp infection.

## Data Availability

The datasets presented in this study can be found in online repositories. The names of the repository/repositories and accession number(s) can be found at: https://www.chictr.org.cn, ChiCTR2200062024.

## Footnotes

^1^ https://www.chictr.org.cn

^2^ https://docs.qiime2.org/2019.4/tutorials/

^3^ https://www.metaboanalyst.ca
